# Incidence and influencing factors of olfactory dysfunction in patients 1 week after endoscopic transsphenoidal resection of pituitary tumor: a cross-sectional study of 158 patients

**DOI:** 10.3389/fneur.2024.1402626

**Published:** 2024-07-16

**Authors:** Sumei Zhou, Zhi Zeng, Min Chen, Linbo Zou, Shirong Shao

**Affiliations:** Department of Neurosurgery, Deyang People’s Hospital, Deyang, Sichuan, China

**Keywords:** pituitary tumor, endoscopic transsphenoidal resection, postoperative olfactory dysfunction, endoscope, influence factor

## Abstract

**Objective:**

To investigate the current situation of olfactory dysfunction in patients after endoscopic transsphenoidal resection of pituitary tumors, and analyze its influencing factors, to provide references for clinical nursing and rehabilitation.

**Methods:**

A cross-sectional study design and convenience sampling method were used to investigate 158 patients with pituitary tumors treated by endoscopic transsphenoidal pituitary tumor resection in the Department of Neurosurgery of three Grade-A general hospitals in Sichuan Province from January 2022 and June 2023. The olfactory function of patients was evaluated 1 week after surgery, and the general clinical data and olfactory related data of patients were collected, and the influencing factors of olfactory disorder were analyzed by logistic regression.

**Results:**

The incidence of olfactory dysfunction was 73.42%. analysis revealed that the formation of blood scabs, nasal cavity adhesion, cerebrospinal fluid leakage and operation time were independent risk factors for olfactory dysfunction in patients after transsphenoidal pituitary tumor resection (*p* < 0.05).

**Conclusion:**

The incidence of olfactory dysfunction is high in patients after endoscopic transsphenoidal resection of pituitary tumors, suggesting that medical staff should pay close attention to and identify patients with olfactory dysfunction based on the guidance of disease knowledge and skills, develop targeted nursing interventions, and promote the improvement of patients’ olfactory function and quality of life.

## 1 Introduction

Pituitary tumor is one of the most common intracranial tumors, with an incidence of 16.7% (1), accounting for 10%–25% of intracranial tumors (2). Endoscopic endonasal transsphenoidal resection has become a popular method for treating pituitary tumors in recent years. This method offers several advantages, including less trauma, fewer complications, and faster recovery (3).

The olfactory nerve fibers pass through the cribriform plate, upper turbinate, upper nasal septum, and middle turbinate. During transsphenoidal pituitary tumor resection, damage to the nasal structures and olfactory nerve is inevitable, which can result in the risk of transient or permanent olfactory loss in patients (4–6). Some scholars have reported that (7), patients who underwent endoscopic surgery experienced a significant decline in nasal integrity 1 month after the surgery. Additionally, 39% of patients had deteriorated olfactory status, and 24.3% experienced anosmia or hyposmia.

Olfactory dysfunction can lead to decreased appetite (8), anxiety, depression, social isolation and other negative emotions (9). It can also significantly impair the ability to detect dangerous odor signals (10), seriously affecting the quality of life and physical health of patients. Currently, some scholars have researched the factors that influence postoperative olfactory dysfunction. However, the included factors are limited, mostly focusing on surgical injuries. There are fewer studies on factors such as the duration of surgery and postoperative nasal cavity condition and other factors (11).

A meta-analysis (12) showed that implementing olfactory nursing interventions during the early postoperative period can effectively improve the degree of dysfunction in patients with pituitary tumor after surgery. Therefore, this study aims to investigate and analyze the factors that influence olfactory impairment in patients 1 week after endoscopic transsphenoidal pituitary tumor resection. The objective is to improve the attention of nursing staff and provide theoretical references for the implementation of targeted interventions as early as possible in clinical practice.

## 2 Materials and methods

### 2.1 Study design and participants

By convenience sampling, we selected pituitary tumor patients who were admitted to the neurosurgery departments of three tertiary general hospitals in Sichuan Province between January 2022 and June 2023.

The inclusion criteria were the following: preoperative cranial imaging (CT or MRI) showed sellar space occupying lesions; postoperative pathology showed pituitary adenoma; first-time pituitary tumor surgery, with the surgical procedure being a neuroendoscopic transsphenoidal pterygoid approach pituitary tumor resection; ≥18 years old; participants had no olfactory dysfunction before operation and could complete the olfactory test; agreed to participate in this study and signed the informed consent.

Exclusion criteria for this study include, nasal septum deviation exceeding 5 mm from the midline; previous cases of head trauma, nasal surgery or radiotherapy; Cases with history of upper respiratory tract infection; subjective olfactory disturbance caused by olfactory sulcus meningioma, tuberculum sellae meningioma and other etiologies; reoperation due to postoperative cerebrospinal fluid leakage, intracranial infection and short-term tumor recurrence; unwillingness to participate in the evaluation of this study or ineffective follow-up.

### 2.2 Ethical considerations

The study approved by the Ethics Committee of Deyang people’s Hospital (No. 2021-04-059-K01), and the personal information of participants was anonymously treated for privacy and confidentiality.

Written informed consent was obtained from all the participants prior to the enrollment of this study.

### 2.3 Research tools

#### 2.3.1 General information survey form

The questionnaire was designed based on the summary analysis of domestic and foreign literature and expert consultation. (i) Demographic sociological characteristics including age, gender, smoking history, drinking history, and allergy history. (ii) Disease-related factors including pituitary tumor size, type, stroke, and operation duration. (iii) Postoperative nasal conditions including blood scab formation, mucosal erosion, nasal adhesion, sinus effusion, and cerebrospinal fluid rhinorrhea. (iv) These underlying diseases included hypertension, diabetes, coronary artery disease, chronic renal disease, chronic obstructive pulmonary disease, etc.

#### 2.3.2 The olfactory dysfunction measuring

The “Five Odour Olfactory Test,” developed by the Institute of Semiconductors, Chinese Academy of Sciences, an odor identification test specifically for the Chinese population (13, 14). The bromine used in the test is a substance that is common in the lives of Chinese people and is also widely used in China. The olfactory test used five standardized odorants: A (acetic acid, which has a sour odor), B (amyl acetate, which has a banana odor), C (eugenol, which has a floral odor), D (menthol, which has a mint odor), E (3-methylindole, which has a fecal odor), and water was the control reagent.

Olfactometry Steps (13, 14): The examiner placed the test solution approximately 1 cm in front of the subject’s nostrils, and asked the subject to sniff 2–3 times without deep inhalation. Five concentrations (labeled 1–5) and five odors (A to E) were tested, starting from the low concentration 1, and the serial number of the concentration when the odor could be felt or recognized was recorded.

Test results (13, 14): At the conclusion of the test, the patients were scored based on their ability to identify the odorant concentration, and the average of the five reagent was considered as the olfactory score, which represented the patient’s olfactory function. Patients were assigned a score of “0” if they could not smell the test liquid at any concentration, and the “0” item was not included in the average calculation. Those with a score of “0” were included in the olfactory dysfunction group. The olfactory function was detected at 1 week postoperatively.

Precautions (13): The olfactory test was conducted 2 h after a meal in all patients who had not taken any medication in the nasal cavity. Smokers need to be tested after 6 h of smoking. When performing the olfactory test, the time interval for replacing the olfactory agent should be 20 to 30 s to avoid olfactory fatigue. The entire process lasted approximately 3–5 min. The test environment needed to be quiet, odorless, well-ventilated, and maintained at a consistent temperature. Both the test subject and the tester were instructed to avoid using any cosmetics or perfumes that contain irritating fragrances on their hands and face.

### 2.4 Data collection

The lead investigator standardized the training for researchers following approval from each hospital and department. After patients provided signed informed consent, trained investigators conducted olfactory assessments and administered general information questionnaires. The completed questionnaires were reviewed immediately and retrieved after confirmation of accuracy. Data entry, de-identification, and analysis were carried out by separate individuals. Two researchers input the questionnaire results, and another member desensitizes the data and submits it to the data analyst. A total of 168 patients were investigated, 10 patients were interrupted due to incorrect olfactory testing, and 158 valid data were finally obtained, with an effectiveness rate of 94.05%.

### 2.5 Statistical analyses

Statistical analyses were conducted using SPSS version 22.0. Continuous variables were described by mean and standard deviation (SD), while categorical variables were summarized by frequencies and percentages. One-way analysis was performed using chi-squared testing. The potential risk factors associated with olfactory dysfunction were first screened through logistic single-factor regression analysis between the two groups. Subsequently, binary logistic multiple-factor regression analysis was introduced to calculate the logistic regression coefficient (B), standard error, Wald value, odds ratio (OR), and the 95% confidence interval (CI) of OR to analyze the independent influences on the occurrence of olfactory dysfunction. A two-sided *p*-value < 0.05 was considered statistically significant.

## 3 Results

### 3.1 Baseline characteristics of the study participants

This study investigated 158 patients who underwent Endoscopic Transsphenoidal Resection of pituitary tumor, 83 (52.53%) were male and 75 (47.47%) were female. Postoperative olfactory dysfunction was observed in 116 patients (73.42%), while 42 patients (26.58%) did not have any issues with their sense of smell.

### 3.2 Univariate analysis of postoperative olfactory dysfunction in patients

The univariate analysis results revealed that (Table 1) age, pituitary adenoma apoplexy, blood scab formation, nasal adhesion, cerebrospinal fluid rhinorrhea, and operation duration were the significant factors that influenced the development of olfactory dysfunction in patients after Endoscopic Transsphenoidal Resection of pituitary tumor (*p* < 0.05).

**Table 1:** Univariate analysis of postoperative olfactory dysfunction (*n* = 158).

	Olfactory dysfunction	No olfactory dysfunction	Statistical value	*p*-value
	(*n* = 42)	(*n* = 116)		
Age			4.823	0.028
<65	27(22.3)	94(77.7)		
≥65	15(40.5)	22(59.5)		
Gender			0.001	0.982
Male	22(26.5)	61(73.5)		
Female	20(26.7)	55(73.3)		
Smoking history			3.111	0.078
Yes	19(35.2)	35(64.8)		
No	23(22.1)	81(77.9)		
Drinking history			0,637	0.425
Yes	20(29.9)	47(70.1)		
No	22(24.2)	69(75.8)		
Allergy history			0.007	0.933
Yes	3(33.3)	6(66.7)		
No	39(26.2)	110(73.8)		
Underlying diseases			0.018	0.893
Yes	14(25.9)	40(74.1)		
No	28(26.9)	76(73.1)		
Pituitary tumor size			0.000	1.000
d < 10 mm	1(50.0)	1(50.0)		
d ≥ 10 mm	41(26.3)	115(73.7)		
Pituitary tumor type			5.026	0.413
Prolactinomas	6(42.9)	8(57.1)		
Somatotropinoma	2(11.8)	15(88.2)		
Adenocorticotroph hormone adenoma	4(25.0)	12(75.0)		
Thyrotropinoma	2(50.0)	2(50.0)		
Luteinizing hormone tumor/Follicle stimulating hormone tumor	5(27.8)	13(72.2)		
Nonfunctioning pituitary adenomas	23(25.8)	66(74.2)		
Pituitary adenoma apoplexy			5.690	0.017
Yes	2(7.7)	24(92.3)		
No	40(30.3)	92(69.7)		
Nasal sphenoid sinus infections			1.785	0.182
Yes	0(0.0)	8(100.0)		
No	42(28.0)	108(72.0)		
Blood scab formation			31.003	<0.001
Yes	12(11.9)	89(88.1)		
No	30(52.6)	27(47.4)		
Mucosal erosion			0.727	0.394
Yes	0(0.0)	5(100.0)		
No	42(27.5)	111(72.5)		
Nasal adhesion			6.315	0.012
Yes	5(11.9)	37(88.1)		
No	37(31.9)	79(68.1)		
Sinus effusion			2.335	0.126
Yes	1(6.7)	14(93.3)		
No	41(28.7)	102(71.3)		
Cerebrospinal fluid rhinorrhea			16.507	<0.001
Yes	5(8.3)	55(91.7)		
No	37(37.8)	61(62.2)		
Operation duration			43.540	<0.001
≥2 h	35(54.7)	29(45.3)		
<2 h	7(7.4)	87(92.6)		

### 3.3 Logistic regression analysis of postoperative olfactory dysfunction in patients

Logistic regression analysis was conducted to determine the relationship between olfactory dysfunction and the independent variables that showed statistically significant results in the univariate analysis (Table 2). The dependent variable was the presence of olfactory dysfunction, denoted as 0 for no dysfunction and 1 for dysfunction. The Hosmer-Lemeshow test (χ^2^ = 7.479, *p* = 0.486 > 0.05) indicated a good model fit. The study revealed that blood scab formation (yes = 0, no = 1), nasal adhesion (yes = 0, no = 1), cerebrospinal fluid rhinorrhea (yes = 0, no = 1), and operation duration (≥2 h = 0, <2 h = 1) were independent risk factors (*p* < 0.05) associated with the development of olfactory dysfunction in patients after Endoscopic Transsphenoidal Resection of pituitary tumor.

**Table 2:** Logistic regression analysis of postoperative olfactory dysfunction in patients (*n* = 158).

Variables	β	SE	Wald x^2^	*P*	OR	95%CI
Constant	0.803	0.531	2.286	0.131	2.232	-
Age	−0.379	0.590	0.414	0.520	0.684	0.215~2.173
Pituitary adenoma apoplexy	0.770	0.863	0.796	0.372	2.161	0.398~11.737
Blood scab formation	1.241	0.497	6.239	0.012	3.457	1.306~9.152
Nasal adhesion	1.527	0.677	5.082	0.024	4.604	1.221~17.364
Cerebrospinal fluid rhinorrhea	1.870	0.625	8.949	0.003	6.490	1.906~22.103
Operation duration	−2.250	0.550	16.704	<0.001	0.105	0.036~0.310

## 4 Discussion

### 4.1 Olfactory dysfunction incidence after transsphenoidal pituitary tumor resection

The study showed that 73.42% of patients had olfactory dysfunction 1 week after endoscopic transsphenoidal pituitary tumor resection. This indicates that olfactory dysfunction is a common occurrence in postoperative patients, and medical staff should monitor changes in olfactory function. The percentage was higher than the findings of Sowerby et al. (15) (40.91%) and Chen (16) (54.84%), and basically consistent with the research results of Wang et al. (17) (75%). The possible reasons are as follows. (i) Different assessment tools. Quantitative measurement of olfactory function include subjective assessment, psychophysical testing, and electrophysiology (18). Patients’ subjective assessment of olfactory function is imprecise and inconsistent with actual olfactory ability due to differences in pain burden and self-esteem (19, 20). Sowerby et al. (15) used the University of Pennsylvania Smell Identification Test (UPSIT), a psychophysical test that requires patients to consciously respond and relate their sensory experience to the properties of a previous physical stimulus. The five-odor olfactory test method used in this study is suitable for assessing olfactory dysfunction in the Chinese population, with a sensitivity of 74% and a specificity of 91.7% (21). While psychophysical tests are considered to produce objective olfactory data, they are not entirely objective and reflect the patient’s subjective interpretation of each odor (22). Therefore, there may be some differences between different assessment tools. (ii) Inconsistency timing of assessment. Sowerby et al. (15) reported the olfactory test results of patients 3 months before surgery, Chen Yanxin (16) reported the occurrence of olfactory dysfunction 6 months after surgery. In contrast, both the present study and Wang et al. (17) selected olfactory assessments 1 week after surgery. During endoscopic transsphenoidal surgery, olfactory nerve fibers may be damaged, resulting in decreased olfactory function. The olfactory cells have a certain repair and regeneration ability, which can be repaired by themselves in about 30 days, and the olfactory damage in most patients can be relieved by themselves in 1–3 months after surgery (23). Some studies have pointed out that (24), the duration of olfactory dysfunction is closely related to the recovery time of olfaction. The longer the duration of olfactory dysfunction, the longer the time required for olfactory recovery. Therefore, inconsistency in the timing of assessment may be one of the factors contributing to the differences in results.

Olfactory dysfunction has been shown to be a common clinical health problem, which has a significant impact on the quality of life of patients (25). In the postoperative nursing process, medical staff should pay early attention to the olfactory situation of patients, and implement early intervention measures to promote the recovery of olfactory function and improve patients’ quality of life.

### 4.2 Influencing factors of olfactory dysfunction after transsphenoidal pituitary tumor resection

#### 4.2.1 Blood scab formation

This study found that the probability of blood scab formation in the nasal cavity of patients after transsphenoidal pituitary tumor resection was 63.92%, which was an important influencing factor of postoperative olfactory dysfunction (*p* < 0.05). Under normal conditions, volatile odor molecules typically reach the olfactory epithelium at the level of the sieve plate, upper nasal septum, and middle/upper turbinate, and activate olfactory receptors by dissolving into the mucus layer (26). After transsphenoidal surgery, the olfactory epithelium and its surrounding mucosa may experience issues such as blood scab formation and structural changes (27), leading to physical blockage of airflow in the nasal cavity, preventing odor molecules from reaching the olfactory receptor neurons and impacting the recovery of olfactory function. Furthermore, eosinophilic infiltration may occur in the area of blood crust formation, resulting in prolonged exposure of the nasal mucosa to fungal extracts, leading to significant thinning of the olfactory epithelium and exacerbating the development of olfactory dysfunction (26, 28, 29). Koskinen et al. (30) also found that the long-standing nasal blood scab may lead to chronic bacterial colonization, and further affecting the olfactory function. It has been concluded that (31), nasal care with saline nasal irrigation and at least 2–3 endonasal debridement procedures within 6 weeks after surgery may shorten the time to olfactory recovery. Nasal irrigation as a common care method to prevent and alleviate nasal complications may be attributed to its physical cleansing mechanism, which has significant advantages in improving mucociliary clearance, controlling local inflammation and preventing mucosal adhesions, and has many advantages such as safety, efficacy, comfort and low healthcare costs (32).

Therefore, it is recommended that medical staff should rinse the nasal cavity and clean the blood scab regularly after surgery to reduce the formation of nasal blood scab, prevent bacterial breeding, and decrease the correlation between blood scab and postoperative olfactory impairment.

#### 4.2.2 Nasal adhesion

The study showed that postoperative nasal adhesions may significantly affect the olfactory function of patients (*p* < 0.05), which is consistent with the results of other studies (33). Studies have shown that (34), patients undergoing endoscopic transsphenoidal surgery have impaired olfactory nerve fibers, increased nasal secretions, and prolonged mucociliary clearance, leading to nasal adhesions. Nasal adhesion causes the olfactory region to be covered by viscous or purulent secretions, which hinders the repair of nasal mucosa and the normal recovery process of olfactory epithelium (18). Olfactins cannot contact with the cilia of olfactory receptor neurons, and the clearance of cilia was impaired, blocking the transmission of odorant to the olfactory mucosa, affecting the perception ability of olfactory cells, and leading to the decline of olfactory function (35). Additionally, Long-standing nasal adhesions have been observed to trigger chronic inflammation of the olfactory epithelium, which further damaged the mucus layer of the respiratory tract and olfactory epithelium, disrupted the normal renewal of olfactory sensory neurons, and interfered with olfactory receptor activation (36). Blood scab formation along the mucosal and olfactory epithelial incisal edge would increase nasal congestion and mucus secretion thickening, prolong the healing time of the nasal mucosa, and affect olfactory function.

As a result of, after the removal of nasal gauze in the postoperative period, it is recommended to perform nasal cleansing and separate the nasal adhesions, enhance the function of nasal mucosal cilia, reduce inflammatory factors, and promote the repair of nasal mucosa and wound healing.

#### 4.2.3 Cerebrospinal fluid rhinorrhea

The study results indicate that patients who experienced cerebrospinal fluid rhinorrhea were 6.490 times more likely to develop olfactory dysfunction than those without (*p* < 0.05). Porras et al. (37) pointed out that postoperative cerebrospinal fluid rhinorrhea is one of the most common complication of transsphenoidal surgery, which has a negative impact on the nasal cavity and olfactory nerves. When cerebrospinal fluid flows into the nasal cavity, the outflow or pulsatile overflow of clear fluid can be observed when its flow is significant. This can lead to physical obstruction in the nasal cavity, interference with the transmission of olfactory molecules and the normal function of the olfactory nerve, and an impact on the odor recognition ability of patients (38). Given the interconnected nature of the nasal cavity and cranial cavity, external bacteria and other pathogens are readily able to invade the central nervous system, leading to intracranial infection and further deterioration of the patient’s condition, which in turn impairs the recovery of olfaction (39). Cerebrospinal fluid rhinorrhea usually requires additional surgery in the naso-pterygoid saddle region, which can further damage the nasal cavity (40).

In the postoperative period, patients should be counseled to avoid risk factors such as strenuous exercise and forceful coughing, as well as refrain from excessive nasal cavity cleansing and the use of tools that may rupture the mucous membranes, as these actions can exacerbate cerebrospinal fluid rhinorrhea. It is recommended that patients adhere to their physician’s instructions and gradually resume their daily activities and exercises within an appropriate time frame.

#### 4.2.4 Operation duration

Studies have shown that the operation duration is significantly correlated with the occurrence of postoperative olfactory dysfunction (*p* < 0.05). The nasal mucosa plays a pivotal role in the respiratory system, and the mucus removal mechanism of its epithelial cilia is highly effective in removing bacteria and crusts from the nasal cavity (41). While endoscopic transnasosphenoidal approach have usually required resection of normal anatomical structures, such as the superior turbinate, middle turbinate, superior septum, or lamina cribrosa, instrument manipulation may damage olfactory nerves and mucosa, which could inevitably affect the sense of smell. The longer the duration of surgery, the longer the nasal dilator compressed the nasal tissues and olfactory nerves (25). Prolonged instrumentation may damage the olfactory nerves, potentially causing more severe tissue edema, congestion, inflammatory response, and olfactory damage, leading to decrease nasal secretions and mucosal atrophy, which could ultimately lead to olfactory dysfunction (42). Some studies have demonstrated that (43), nasal mucosal edema can further impede the transport of olfactory molecules to receptor neurons, thereby interfering with the transmission of odor molecules and the perception of olfactory signals, which can result in olfactory decline.

In addition, it is important to handle the tissues with care and minimize trauma to the nasal cavity in order to reduce the inflammatory response.

## 5 Conclusion

In view of the increasing concern about the quality of life of patients, the olfactory outcome after transsphenoidal surgery has been a neglected research area. The study revealed that the incidence of olfactory dysfunction in patients after endoscopic transsphenoidal pituitary tumor resection was higher, and the main influencing factors included blood scab formation, nasal adhesion, cerebrospinal fluid rhinorrhea, and operation duration. Some studies have indicated that (12), olfactory nursing intervention following surgery can activate olfactory epithelial stem cells in the nasal cavity, promote the recovery of olfactory mucosa and olfactory nerve, improve the sensitivity of odor recognition and discrimination, reduce olfactory threshold score, and promote the recovery of olfactory function. In clinical practice, medical staff are aware of the significance of olfactory dysfunction and integrate subjective and objective olfactory assessment into the standard postoperative treatment regimen. They promptly identify olfactory dysfunction and its risk factors and implement appropriate intervention measures, such as nasal irrigation, olfactory stimulation, and training, which can enhance nasal function scores and mitigate the severity of olfactory dysfunction (35).

This study recommends that, saline nasal irrigation should be initiated 24 h after the removal of bilateral nasal packing to clean the nasal cavity and separate nasal adhesions, improve the function of nasal mucosa cilia, reduce inflammatory factors, and promote the repair of nasal mucosa and wound healing. Nevertheless, high-volume irrigation may result in scabs and the separation of the reconstructed skull base, which can lead to significant nosebleeds and cerebrospinal fluid leakage, and has poor safety. Therefore, a nasal spray with a small rinsing ability should be used, with three to six compressions per nostril, with each rinsing requiring simultaneous rinsing of both sides of the nasal cavity, 5–6 times a day in the first month after surgery, 3–4 times a day in the second month, and 1–2 times a day in the third month (32).

Olfactory training is a therapeutic approach that enhances olfactory function by exposing subjects to different odors at regular intervals (44). This approach is beneficial not only for the treatment of patients with olfactory disorders, but also for the enhancement of olfactory ability in healthy individuals. One week following surgery, patients underwent nasal decongestion and were subsequently administered olfactory training with four distinct odors (phenylephrine: rose, eucalyptol: eucalyptus, citromellal: lemon, and eugenol: eugenol) within a ventilated, odor-free environment. Patients were instructed to smell each bromides for 10s each time, and the interval between the two bromides was 10 s (45).

The study has some limitations, such as a low sample representativeness and no follow-up observation of the patients’ long-term recovery from olfactory dysfunction. It is recommended that future studies should carry out larger samples to verify the reliability and popularization of the results and extend the observation time to analyze the longitudinal change trend of the patients’ olfactory function.

## Data availability statement

The original contributions presented in the study are included in the article/Supplementary material, further inquiries can be directed to the corresponding author.

## Ethics statement

The studies involving humans were approved by the Ethics Committee of Deyang people’s Hospital. The studies were conducted in accordance with the local legislation and institutional requirements. The participants provided their written informed consent to participate in this study.

## Author contributions

SZ: Conceptualization, Data curation, Project administration, Writing – original draft, Writing – review & editing. ZZ: Methodology, Visualization, Writing – original draft, Writing – review & editing. MC: Data curation, Investigation, Methodology, Supervision, Validation, Visualization, Writing – review & editing. LZ: Formal analysis, Methodology, Writing – review & editing. SS: Funding acquisition, Supervision, Writing – review & editing.
